# Post-Fever Retinitis

**DOI:** 10.7759/cureus.26429

**Published:** 2022-06-29

**Authors:** Pranaykumar Shinde, Sachin Daigavane, Avi Sharma

**Affiliations:** 1 Ophthalmology, Jawaharlal Nehru Medical College, Sawangi (Meghe), Wardha, IND

**Keywords:** triamcinolone acetonide, fundus fluorescein angiography (ffa), oct (optical coherence tomography), post fever, retinitis

## Abstract

A 34-year-old male presented with a gradual, painless decrease in distant vision in the left more than the right in the past 15 days. He had a history of an episode of fever seven days prior to ocular symptoms and had been treated with oral Cefixime and antipyretics. On detailed fundus examination, both eyes showed creamy white superficial lesions with ill-defined margins, suggestive of retinitis, with a few hemorrhages along the inferior arcade associated with sheathing of vessels and telangiectatic vessels near the lesion. Fundus fluorescein angiography (FFA) of both eyes in the venous phase depicted blocked fluorescence at the site of retinitis lesions with hyperfluorescence at their borders. Optical coherence tomography (OCT) of both eyes showed inner retinal layer hyper-reflectivity with neurosensory detachment (NSD) with hyper-reflective deposits in it and dilated choroidal vessels. The diagnosis of both eyes' post-fever retinitis (PFR) was made, and he was investigated for complete blood count, peripheral smear, erythrocyte sedimentation rate, and HIV Tridot test, which were normal. He was treated with intravitreal triamcinolone acetonide (2 mg/0.05 ml) in both eyes.

## Introduction

The term post-fever retinitis (PFR) is used to describe various retinal manifestations following any systemic febrile illness, which can be due to bacteria, viruses, or protozoa. It usually manifests within two to four weeks post fever in immunocompetent patients irrespective of etiology [1].

It is either due to direct invasion by a pathogen or indirectly mediated through an immune-mediated mechanism and usually presents with a sudden diminution of vision.

## Case presentation

**Figure 1:**
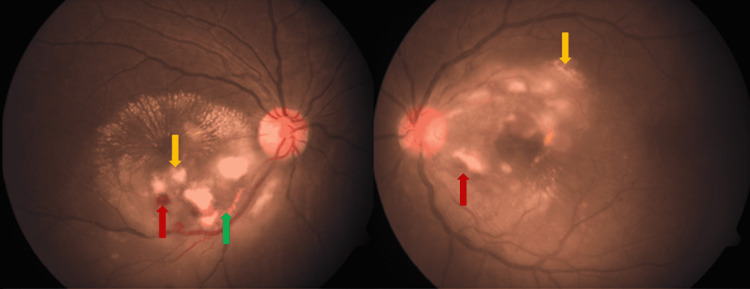
Color fundus picture of both eyes showing clear media, normal disc, yellow arrow showing retinitis lesions in both the eyes. Intraretinal hemorrhages in both eyes shown by red arrows. Green arrow showing perivascular sheathing in right eye. Also inflammatory telangiectatic vessels can be appreciated at the border of retinitis lesions in both eyes.

A 34-year-old male presented with a gradual decrease in distant vision in both eyes, left more than right, for 15 days, which was painless. He gave a history of fever seven days prior to ocular symptoms and had been treated with oral Cefixime and antipyretics. The fever was intermittent in nature and subsided with antipyretics. No history of rash, respiratory symptoms, a burning sensation on micturition, or joint pain. The right eye and left eye vision were 20/60 and counting finger 2 meters, respectively. The intraocular pressure in the right eye was 15 mm of Hg and in the left eye was 16 mm of Hg, as recorded by applanation tonometry. Anterior segment findings were unremarkable with a clear lens in both eyes. On detailed fundus examination, the right eye showed clear media with a normal disc. There were creamy white superficial lesions with ill-defined margins suggestive of retinitis, with a few hemorrhages along the inferior arcade associated with sheathing of vessels and telangiectatic vessels near the lesion. We could appreciate the hard exudate plaque at the fovea and also surround it, forming a macular star. A similar kind of lesion was present in the left eye posterior pole associated with hemorrhages, sheathing of vessels, telangiectatic vessels, and a few hard exudates (Figure 1).

**Figure 2:**
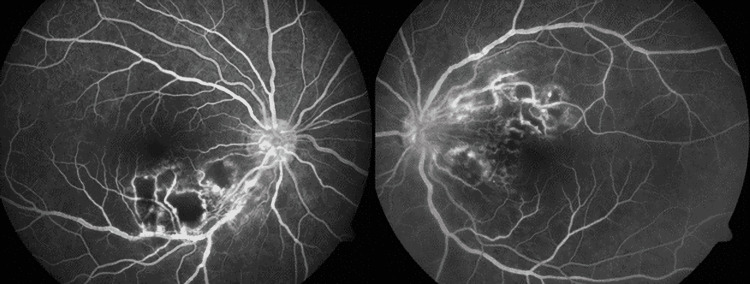
Both eyes fundus fluorescein angiography in venous phase showing blocked fluorescence at the site of retinitis lesions with hyperfluorescence at its borders and around sheathing vessel.

Fundus fluorescein angiography (FFA) of both eyes in the venous phase depicted blocked fluorescence at the site of retinitis lesions with hyper-fluorescence at their borders and also around the sheathing vessel (Figure 2).

**Figure 3:**
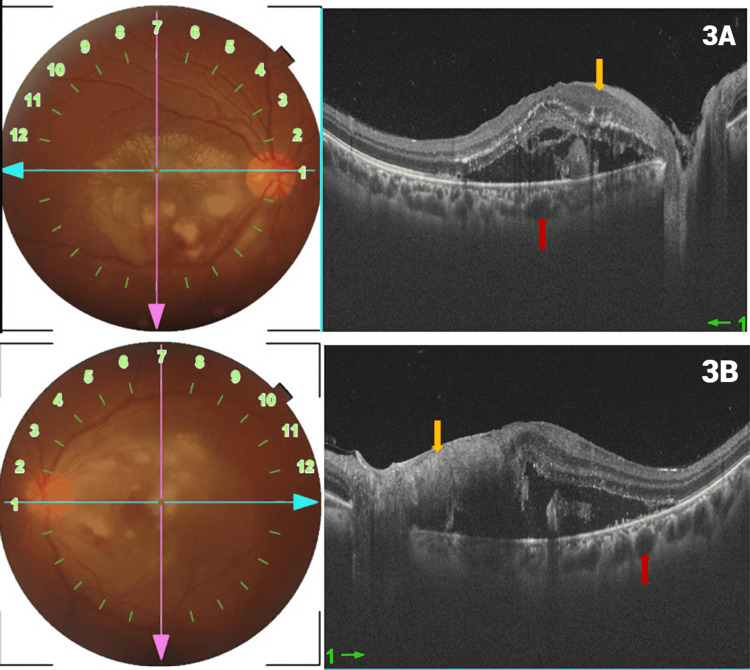
(A) and (B) shows horizontal optical coherence tomography scans passing through disc and macula depicts inner retinal layer hyper-reflectivity (yellow arrow), neurosensory detachment, containing hyper-reflective material and pachychoroid (red arrow).

Optical coherence tomography (OCT) of both eyes showed inner retinal layer hyper-reflectivity with neurosensory detachment (NSD) with hyper-reflective deposits in it and dilated choroidal vessels (Figure 3A-3B).

The diagnosis of both eyes post-fever retinitis was made and he was investigated for complete blood count, peripheral smear, erythrocyte sedimentation rate, C-reactive protein, malaria, Dengue IgM and IgG, Widal test, Mantoux test, urine routine, and HIV Tridot test, which were within normal limits.

**Figure 4:**
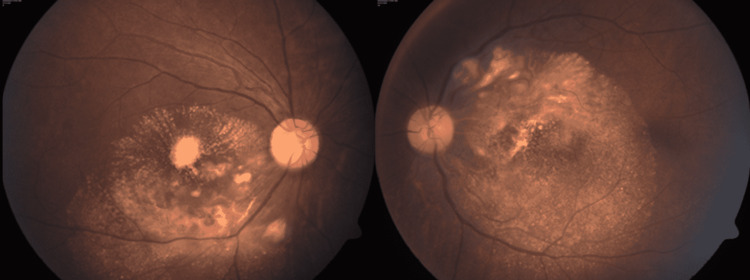
Color fundus photo of both eye showing resolving retinitis lesions with delineated margins as compared to previous visit after one dose of intravitreal Triamcinolone Acetonide.

He was advised to inject both eyes with intravitreal triamcinolone acetonide (2 mg/0.05 ml). Two weeks later, the vision in the right eye had improved to 20/100 and in the left eye was 20/600. The margins of retinitis lesions looked more delineated with the appearance of new telangiectatic vessels in both eyes (Figure 4).

**Figure 5:**
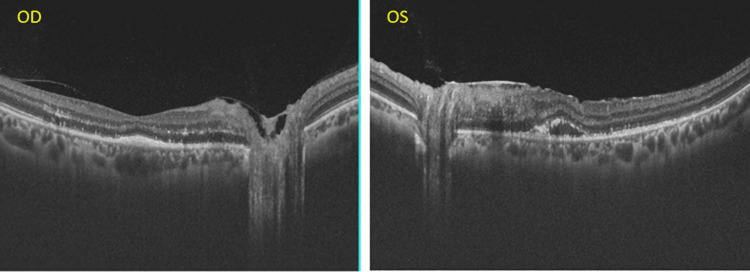
Optical coherence tomography scans of both eyes passing through disc and macula shows resolving neurosensory detachment with restoration of retinal layers.

OCT of both eyes revealed resolving NSDs with the restoration of retinal layers. He was then kept on follow-up (Figure 5).

## Discussion

PFR can be present with focal or multifocal retinitis and can be unilateral or bilateral. Also, anterior uveitis, macular edema, and serous retinal detachment at the macula can be manifested. Localized vessel involvement in the form of beading, tortuosity, and perivascular sheathing are other striking features. FFA in PFR reveals early hypofluorescence, late hyperfluorescence, disc leakage, and localized staining of vessels. OCT imaging reveals nerve fiber layer hyperreflectivity with after-shadowing in retinitis areas, as well as cystic spaces in the outer retina and serous detachment at the macula [2].

Based on our experience and the review of literature, we suggest that post-fever retinitis and vasculitis follow a natural course in which the symptoms first arise, culminating in a peak of progression, and then resolve. The natural courses follow a bell curve. Manifestations start to progress to their peak and then terminally resolve [1].

Rickettsia, West Nile virus, chikungunya, dengue, and Zika virus are bacterial causes of PFR that are treated with systemic antibiotics and possibly systemic steroids. Viral causes of PFR, such as chikungunya, dengue, and West Nile virus, however, do not have a specific treatment and are managed with steroids. Polymerase chain reaction (PCR) analysis of aqueous to rule out any infectious etiology helps to avoid exacerbations of nonimmune-mediated retinitis. Good response to steroids irrespective of etiology indicates a possible immunological role in this condition. A study of this entity aided by histopathological and immunological aids will help to gain a deeper understanding of the pathophysiology.

## Conclusions

Post-fever retinitis can be associated with inflammatory telangiectatic vessels that subside with the resolution of inflammation. This case demonstrates the resolution of immune-mediated retinitis following an episode of fever. The exact etiology of fever and retinitis could not be ruled out. A viral etiology cannot be completely ruled out.

The lesions, in this case, were central creamy white superficial lesions with ill-defined margins, suggestive of retinitis, with few hemorrhages along the inferior arcade associated with sheathing of vessels and telangiectatic vessels near the lesion, did not exhibit circumferential spread, and did not exhibit prominent anterior or posterior cellular reaction. Though this kind of retinitis is preceded by fever, differential diagnosis of other causes of retinitis should always be kept in mind.
